# 

**Published:** 1867-11

**Authors:** 


### THE CHICAGO MEDICAL JOURNAL.

**Edited by J. ADAMS ALLEN, M.D., LL.D.**

Prof. Principles and Practice of Medicine and Clinical Medicine in Rush Med. College.

All letters for the JOURNAL should be addressed to DR. J. ADAMS ALLEN, P.O. Box 1948, Chicago, Ill.

**TERMS---$2 PER ANNUM, IN ADVANCE.**

$3 if not paid before 1st of July in each year. Single copies, 25 cents.

*The Practice of Medicine and Surgery, Applied to the Diseases*

*and Accidents incident to Women.* By WM. H. BYFORD, A.M., M.D., author of "A Treatise on the Chronic Inflammation and Displacements of the Unimpregnated Uterus;" and Professor of Obstetrics and Diseases of Women and Children in the Chicago Medical College. Second edition, enlarged. Philadelphia: Lindsay & Blakiston. 1867.

*A Biennial Retrospect of Medicine, Surgery, and their Allied Sciences.* Edited by Mr. H. POWER, Dr. ANSTIE, Mr. HOLMES, Mr. THOMAS WINDSOR, Dr. BARNES, and Dr. C. HILTON FAGGE, for the New Sydenham Society. Philadelphia: Lindsay & Blakiston. 1867.

*Inhalation, its Therapeutics and Practice:* A Treatise on the Inhalation of Gases, Vapors, Nebulized Fluids, and Powders, including a description of the Apparatus employed, and a record of numerous experiments, physiological and pathological, with cases. By J. SOLIS COHEN, M.D. Illustrated. Philadelphia: Lindsay & Blakiston. 1867.

*Studies in Pathology and Therapeutics.* By SAMUEL HENRY DICKSON, M.D., LL.D., Professor of Practice of Physic in Jefferson Medical College, Philadelphia, etc. New York: Wm. Wood & Co., 61 Walker St. 1867.

*Headaches: Their Causes and Their Cure.* By HENRY G. WRIGHT, M.D., M.R.C.S.L., L.S.A., Member of the Royal College of Physicians of England, etc., etc. From the fourth London edition. Philadelphia: Lindsay & Blakiston. 1867.

*Epidemic Meningitis, or Cerebro-Spinal Meningitis.* By ALFRED STILLE, M.D., Professor of Theory and Practice of Medicine and Clinical Medicine in the University of Pennsylvania, etc. Philadelphia: Lindsay & Blakiston. 1867.

*Hufeland's Art of Prolonging Life.* Edited by ERASMUS WILSON, F.R.S., author of "A System of Human Anatomy," Diseases of the Skin, etc. From the last London edition. Philadelphia: Lindsay & Blakiston. 1867.

*Circular No. 7.* War Department, Surgeon-General's Office, Washington, July 1st, 1867. A Report on Amputations at the Hip-Joint in Military Surgery. Washington: Government Printing Office. 1867.

